# Using Acoustic Fields to Fabricate ECM-Based Biomaterials for Regenerative Medicine Applications

**DOI:** 10.21926/rpm.2003018

**Published:** 2020-07-21

**Authors:** Emma G. Norris, Diane Dalecki, Denise C. Hocking

**Affiliations:** 1. Department of Pharmacology and Physiology, University of Rochester, Rochester, New York, USA;; 2. Department of Biomedical Engineering, University of Rochester, Rochester, New York, USA;

**Keywords:** Ultrasound, extracellular matrix, collagen, acoustics, biofabrication

## Abstract

Ultrasound is emerging as a promising tool for both characterizing and fabricating engineered biomaterials. Ultrasound-based technologies offer a diverse toolbox with outstanding capacity for optimization and customization within a variety of therapeutic contexts, including improved extracellular matrix-based materials for regenerative medicine applications. Non-invasive ultrasound fabrication tools include the use of thermal and mechanical effects of acoustic waves to modify the structure and function of extracellular matrix scaffolds both directly, and indirectly via biochemical and cellular mediators. Materials derived from components of native extracellular matrix are an essential component of engineered biomaterials designed to stimulate cell and tissue functions and repair or replace injured tissues. Thus, continued investigations into biological and acoustic mechanisms by which ultrasound can be used to manipulate extracellular matrix components within three-dimensional hydrogels hold much potential to enable the production of improved biomaterials for clinical and research applications.

## 1. Introduction

Tissue engineering combines the methodologies of cell biology, chemistry, and engineering to produce materials that replace or facilitate the repair of diseased or injured tissue [1]. Tissue engineering techniques typically utilize progenitor cells, biological scaffolds, and bioactive molecules, either alone or in combination, to achieve desired tissue characteristics [1, 2]. While the potential of tissue engineering to produce laboratory-grown, whole-organ transplants has gained widespread attention [2], other applications of tissue engineering include the development of materials that facilitate endogenous tissue repair [3]. Tissue engineering also offers the opportunity to address fundamental biological and physiological questions that cannot be answered with conventional approaches through the development of artificial tissues or organs for research purposes, as typified by “lab-on-a-chip” technologies [4].

The extracellular matrix (ECM) is a complex network of fibrillar glycoproteins and associated molecules that serves the critical role of defining tissue structure while also providing key biochemical and mechanical cues [5]. Cell-mediated assembly of ECMs plays essential roles in wound healing in many tissues [6, 7], and in tissue assembly during embryonic development [5]. As such, it is no surprise that therapeutic biomaterials targeting ECM assembly, structure, and function have become essential components from which to develop biomaterials for regenerative medicine applications. The potential clinical impact of ECM-based biomaterials is far-reaching, with utility for the treatment of injuries in tissues with low regenerative potential such as peripheral nerves [8], as well as chronic wounds in which the normal progression of healing is dysregulated [9]. In spite of significant progress, limitations still persist in the ability to engineer biomaterials that sufficiently recapitulate critical features of cell-assembled ECMs produced by healthy tissue [2, 10]. Thus, a critical need remains for technologies that can close the gap between the complex, highly organized structures of native ECMs and engineered products designed to enhance tissue repair.

Ultrasound is a versatile biomedical tool that has already revolutionized multiple domains of modern health care with non-invasive approaches to both diagnosis and therapy [11, 12]. Applications of ultrasound for tissue engineering have expanded rapidly [13], and include important contributions to both the characterization [14] and fabrication of engineered tissues [10]. In this review, we present recent innovations in the application of acoustic methodologies to manipulate both materials constructed from native ECM proteins, and synthetic scaffolds designed to mimic structural and functional aspects of the ECM in tissue homeostasis and repair. These applications include the strategic use of ultrasound to alter ECM protein structure via thermal effects and/or mechanical forces, as well as the use of engineered, acoustically responsive elements to manipulate scaffold properties. Finally, we discuss the use of ultrasound to enhance cell-mediated ECM remodeling behaviors, thereby indirectly modifying engineered scaffold structure. Together, these emerging ultrasound-based methodologies offer innovative strategies to enable non-invasive manufacturing and in situ translation of therapeutic materials for regenerative medicine.

## 2. Tissue Injury and Regeneration

Tissue repair in response to injury is an integral part of the physiology of many adult tissues but may be impaired as the result of comorbid health conditions. As well, the inherent repair capacity of some tissues is low. In both cases, failure to repair damaged tissue often leads to lifelong disability or premature death. Thus, interventions that support restoration of tissue function are of vital clinical relevance. In this section, we use dermal wound healing as a representative example to illustrate functional roles and clinical impacts of the ECM in tissue repair. It is important to note that the assembly, composition and organization of ECM structures are tissue-specific, and vary during course of development, with adult homeostasis, and during tissue repair in response to injury [15]. However, given the central role that ECMs play in organizing tissue structure and controlling function [5], the principles described herein apply to a variety of different tissues. Indeed, similar approaches for incorporating ECM-derived, tissue-specific cues into engineering frameworks have recently been reviewed for the treatment of injuries in bone [16], cartilage [17], tendon [18], cornea [19], muscle [20], and peripheral nerves [8, 21], and have been discussed extensively for chronic wounds [9, 22].

### 2.1 Role of Cell-Mediated ECM Remodeling in Wound Healing

Wound healing in response to injury relies on coordinated processes to rapidly induce hemostasis, followed by construction of replacement tissue [6]. Throughout this process, the ECM plays a critical role as a structure- and function-defining scaffold that coordinates cell and tissue responses [9]. Immediately following cutaneous injury, a transient ECM consisting primarily of fibrin and fibronectin forms quickly via the clotting cascade to restore homeostasis and barrier function [23]. This “provisional matrix” also supports subsequent stages of wound healing, including recruitment of immune mediators as well as activated fibroblasts from the surrounding tissue [24]. Cells that migrate into the wound space contribute to ongoing ECM remodeling through coordinated processes of matrix degradation, synthesis, and contraction [6, 25]. Several key signaling mechanisms are involved in the translation of ECM remodeling into coordinated tissue responses. These include cell-derived tensile forces and subsequent matrix contraction, which exposes cryptic, bioactive epitopes in matrix proteins [26], provides topological cues such as fiber alignment [27], and increases tissue tensile strength [28]. Matrix remodeling also acts as a cue for subsequent steps of tissue regeneration, as cell-derived tensile forces and associated changes in the surrounding ECM are key factors in recruitment and function of numerous cell types, including fibroblasts [29, 30], macrophages [31], and vascular cells [32]. In addition, many ECM components contain binding sites for growth factors, serving to sequester or present growth factors to cells in appropriate conformations [33]. Matrix remodeling and cell signaling are also influenced by proteolytic activity, particularly via activation of matrix metalloproteases (MMPs) and associated regulatory proteins (TIMPs), which are key facilitators of ECM turnover and release of soluble signaling factors [34].

### 2.2 ECM Dysfunction in Chronic Wounds

The collective result of these coordinated remodeling processes is the generation of replacement tissue in which the primary ECM components are types I and III collagen [6]. Although healed skin never reaches the full integrity of uninjured tissue, successful wound healing produces a stratified structure that sufficiently replaces the physiological function and mechanical integrity of uninjured skin tissue [35]. In contrast, failure of the wound healing process is associated with a number of chronic conditions, including obesity, diabetes, and peripheral vascular disease, many of which disproportionately affect aging populations [36, 37]. As a result, chronic and non-healing wounds are a widespread public health burden affecting an estimated 20 million people worldwide [38]. While the underlying etiologies of chronic wounds are complex and multifactorial, dysfunctional ECM remodeling may contribute to a number of wound healing pathologies. Long-term (> 20 years) diabetes is associated with increased glycation of dermal collagen [39]. In vitro investigations into downstream effects of collagen glycation have identified changes in fibroblast remodeling behavior, including decreased fibronectin matrix deposition and collagen gel contraction [40], as well as decreased proliferative capacity in endothelial cells [41]. Abnormal quantities and distribution of numerous ECM components have also been observed in histological sections taken from diabetic and venous ulcers in human patients [42, 43].

As impaired matrix remodeling has been associated with wound healing defects, cell-assembled matrices are emerging as valuable tools to treat non-healing wounds. Cell-remodeled collagen scaffolds were among the first engineered biomaterials to receive FDA approval and are still in use in today’s clinical environments [2, 44]. Critically, investigations of donor cell persistence within such materials suggest that donor cells are not maintained past 4 weeks post-transplantation [45]. This suggests that mechanisms by which cell-embedded biomaterials facilitate healing are not dependent on the persistence of donor cells, but rather the ability of cell-remodeled matrices to support host cell infiltration and subsequent healing [3]. The broad utility of cell-derived ECM as a regenerative template is further exemplified by the use of decellularized xenogenic or allogenic ECMs for a diverse array of regenerative medicine applications [44, 46]. In these applications, tissues such as porcine small intestinal submucosa or cadaveric tissue are decellularized, sterilized, and lyophilized to prepare acellular scaffolds comprised of a heterogeneous mixture of ECM proteins [47], glycosaminoglycans [48], and growth factors [49]. Decellularized ECM scaffolds are either in clinical use or under investigation as therapeutics for a broad spectrum of regenerative medicine applications, including vascular, urinary, skin, and nerve reconstruction [8, 46].

In summary, the ECM performs key structural and signaling functions throughout the wound healing process. Cell-mediated ECM remodeling via mechanical contraction, proteolysis, and matrix deposition directs cell signaling through exposure of neoepitopes, growth factor release, and topographic cues. The unique functional contributions of each of these components varies among tissues, injuries, and disease states. Yet, the versatility of decellularized tissue as a regenerative biomaterial suggests that many of these mechanisms are common to a variety of wound healing and regeneration processes. As such, incorporating key structural and functional characteristics of cell-remodeled ECMs into the design of therapeutic biomaterials is a promising strategy for facilitating tissue repair.

## 3. Biological Effects of Ultrasound

Biomedical ultrasound has been employed for a variety of diagnostic and therapeutic applications. Ultrasound is defined as sound with frequencies above the upper limit of human hearing (20 kHz), with current FDA-approved ultrasound devices operating up to 20 MHz [11–13]. Higher frequency devices are also used for a variety of applications, including acoustic microscopy [12]. Diagnostic ultrasound has an unparalleled safety record in comparison with other imaging modalities, many of which rely on ionizing radiation or hazardous contrast agents to acquire images [50, 51]. Extensive work devoted to understanding the interaction of sound waves with tissue, and the potential for biological effects, has been essential to establishing safety guidelines for diagnostic applications of ultrasound in clinical settings [51].

A growing number of ultrasound applications employ the deliberate induction of ultrasound bioeffects for therapeutic benefit. These techniques include well-established therapies such as shock wave lithotripsy, as well as non-invasive surgery using high-intensity focused ultrasound (HIFU) fields [11]. A new and rapidly expanding application is the use of ultrasound for tissue engineering and regenerative medicine. Ultrasound-based tissue engineering technologies have already yielded a number of innovative approaches for using ultrasound to organize cells, proteins and microparticles within in vitro environments [13]. In the following section, a brief overview of the known biological effects of ultrasound is presented. Several representative examples then follow to illustrate the wide range of strategies that have been employed to harness ultrasound for the purposes of producing biomaterials with enhanced functionality.

### 3.1 Sound Propagation Through Tissue

Propagation of sound waves through a medium results in a decrease in acoustic amplitude due to acoustic attenuation. In tissue, sound attenuation occurs via both scattering of sound by heterogeneous tissue structures, as well as direct absorption of acoustic energy [52, 53]. The attenuation coefficient (α) describes the rate at which sound energy is lost in a material over a given propagation distance and is dependent upon the frequency of the sound field. Typical values for the acoustic attenuation coefficient in solid tissue at 1 MHz frequency range from approximately 1 dB/cm in some soft tissues to upwards of 10 dB/cm for highly attenuating tissues such as bone [54]. Absorption of sound by tissue arises from the absorption behavior of its biochemical components, as well as macromolecular interactions between structures such as cells and proteins [55, 56]. The absorption properties of many proteins in dilute solutions have been characterized, and generally increase as a function of protein concentration, acoustic frequency [57, 58], and the extent of chemical crosslinking and other intermolecular interactions [55, 56].

As the most abundant protein in the body, the acoustic properties of collagen are of particular relevance for understanding the acoustic properties of tissue. However, technical limitations arising from the high viscosity and self-assembly capacity of purified collagen solutions have made quantitative characterization of collagen at physiologically-relevant concentrations infeasible [58]. Characterization studies of dilute collagen suspensions in acidic solution have demonstrated that collagen exhibits relatively high absorption in comparison to globular proteins at comparable concentrations and acoustic frequencies [58, 59]. Further, the fibrillar structure of collagen is thought to be a contributor to the acoustic scattering behavior of solid tissues [52, 60]. Recent work has demonstrated that changes in collagen fiber structure, due to concentration and polymerization temperature, influence the amount of backscattered acoustic signal in quantitative ultrasound imaging applications [61]. These results are consistent with reports of increased acoustic attenuation as a result of chemical crosslinking [56], however detailed investigations of how the acoustic properties of collagen change as fibers are formed have not been published.

### 3.2 Acoustic Mechanisms of Ultrasound-Induced Bioeffects

Absorption of sound in tissues can result in several biological effects with clinical relevance. First, significant heating may arise from the absorption of acoustic energy, and the extent of heating is dependent upon the acoustic exposure intensity, acoustic frequency, as well as the absorption properties of the propagation medium [53]. In therapeutic applications, HIFU fields have been used as a non-invasive surgical technique, in which acoustic beam forming methods selectively heat a small focal area without damage to the intervening tissue [11]. HIFU treatments have been FDA-approved for ablation of uterine leiomyomas, bone metastases, and prostate cancer, with many additional applications currently in different stages of clinical trials [62].

Sound propagation through a medium is also associated with a radiation pressure in the direction of acoustic propagation [63]. In solid materials, this pressure results in the generation of an acoustic radiation force and is of particular interest to ultrasound elastography applications, such as acoustic radiation force impulse (ARFI) imaging [64]. In fluids, the same radiation pressure results in fluid flow in the direction of acoustic propagation, referred to as acoustic streaming [65]. In experimental and simulation studies of streaming within cylindrical wells, such as those found in typical tissue culture systems, this includes the generation of cylindrical vortices around the axis of acoustic propagation [66]. Ultrasound-driven fluid streaming has been used to enhance fluid mixing in a variety of applications, including in vitro biological culture systems [67, 68].

In addition to radiation forces associated with travelling wave fields, interaction of multiple waves can result in the generation of ultrasound standing wave fields (USWF), which are characterized by stable regions of zero pressure (nodes) and pressure maxima (antinodes) that result from interference patterns between interacting fields [69]. Particles suspended within a standing wave field are subject to radiation forces, causing particles to cluster at either nodal or antinodal locations [70]. Radiation forces associated with USWFs are the primary mechanism for a number of applications in which ultrasound has been used to distribute particles, including cells and microbubbles into pre-defined patterns [13]. The use of ultrasound to non-invasively pattern cells is of growing interest to tissue engineering, as spatial cues such as relative position, spacing, and density of cells serve as important determinants of cellular behavior [71–73]. USWF exposures have been used to pattern a variety of cell types, including fibroblasts, endothelial cells, Schwann cells, and myocytes to produce enhanced collagen gel contraction [74], vascular network formation [75–77], and cellular alignment [78–81], respectively.

In addition to absorption-dependent mechanisms of ultrasound bioeffects, pressure oscillations associated with acoustic waves can cause expansion and compression of gas bubbles within both fluids and tissues [82, 83]. Bubble oscillations of small amplitude around their equilibrium size are known as stable (or non-inertial) cavitation [84, 85]. Stabilized microbubbles are widely used to enhance contrast during ultrasound imaging, particularly in cardiovascular applications [86]. In contrast, ultrasound-induced expansion of a gas bubble to several times its initial radius can cause inertial collapse of the microbubble. This effect, known as inertial or transient cavitation, produces extremely high pressures, temperatures, and fluid velocities with the potential to damage biological structures [83, 87]. Numerous applications of inertial cavitation are also under investigation for therapeutic applications, including drug delivery, clot lysis, and gene transfection through sonoporation techniques [88].

## 4. Acoustic Manipulation of ECM-Based Scaffolds

**Figure 1:**
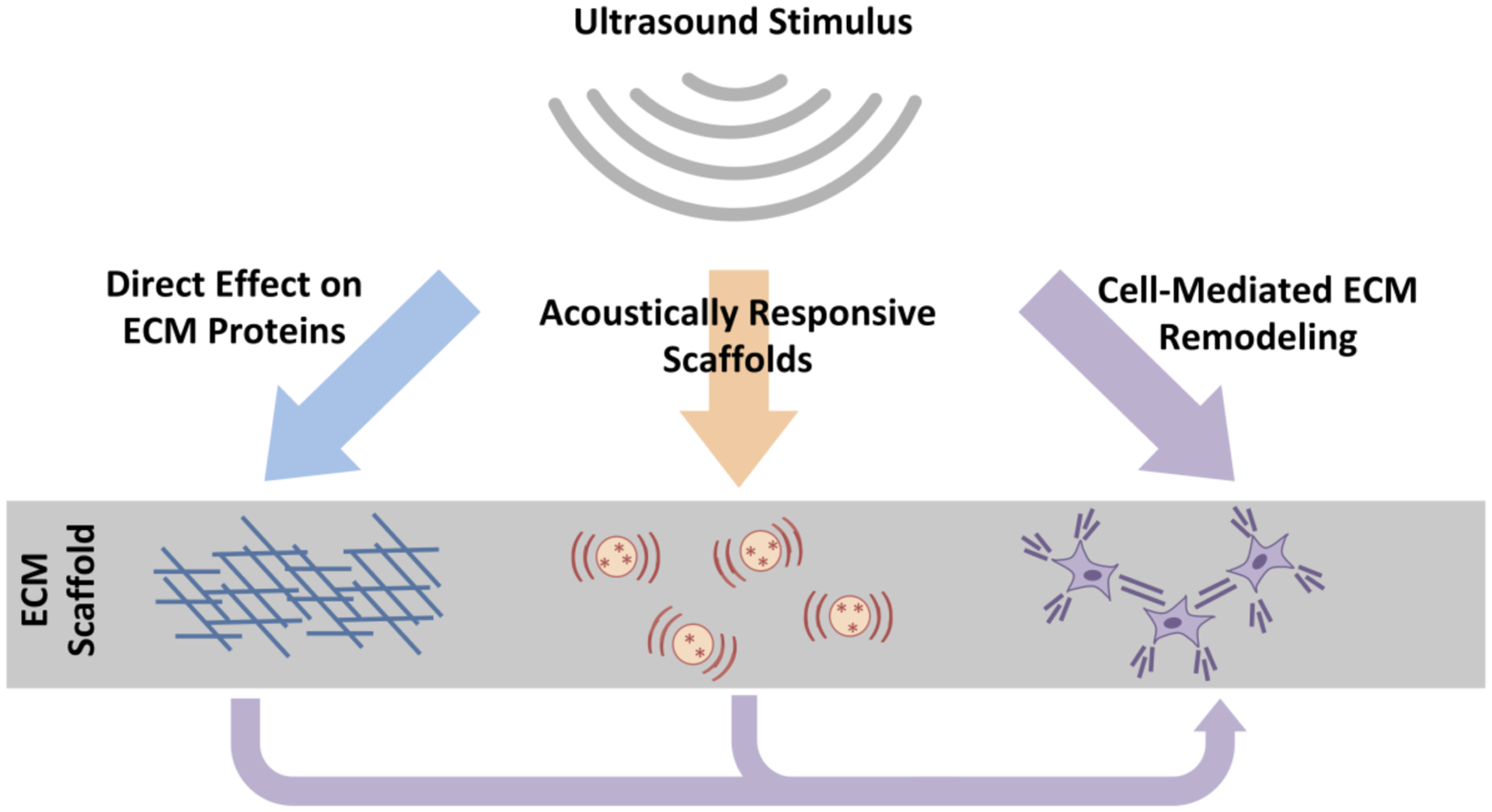
Mechanisms of Ultrasound-ECM Interactions. Ultrasound exposure parameters can be tuned to affect ECM proteins directly via heating, radiation force, or microbubble cavitation. Scaffolds can also be engineered with acoustically responsive elements for drug and protein delivery with spatial and temporal resolution. Additionally, mechanical forces associated with ultrasound may induce cell remodeling activity directly, or indirectly via changes in ECM protein composition or conformation.

The ability of ultrasound to exert non-invasive, spatially- and temporally-localized effects within biological systems makes it an attractive tool for tissue engineering [13]. In particular, native protein components of the ECM are inherently sensitive to both temperature and mechanical forces [89–91], both of which can be induced non-invasively by ultrasound exposures of sufficient intensity [13, 92]. The versatility of ultrasound-mediated effects on ECM-based scaffolds has been furthered by the incorporation of thermal- or cavitation-responsive elements into ECM-based scaffolds to provide acoustic sensitivity. Finally, within the context of cell-embedded biomaterials, ultrasound may also indirectly influence ECM structure and function by stimulating cell behaviors involved in ECM remodeling. Together, these ultrasound-based technologies hold great potential for optimizing and customizing ECM-based biomaterials for regenerative medicine by non-invasively and site-specifically tuning their mechanical, chemical, and biological properties (Figure 1). In the remainder of this review, we discuss these approaches, with representative examples highlighted in Table 1.

### 4.1 Direct Effects of Ultrasound on ECM Proteins

Early investigations into effects of ultrasound on ECM structure and function demonstrated that exposing fibrin clots to ultrasound (0.2 – 4 MHz, 1–8 W/cm^2^) in the presence of proteolytic enzymes (i.e., tissue plasminogen activator, tPA) accelerated the rate of fibrin degradation [94, 105]. Subsequent mechanistic investigations demonstrated that ultrasound did not fragment fibrin scaffolds directly [94]. Rather, in the presence of ultrasound, fibrin fibers were separated into strands of reduced diameter and local density [95]. While the overall fiber structure of the fibrin gels returned to its pre-exposure state once ultrasound was removed, the transient reduction in fiber density was sufficient to accelerate proteolytic degradation [94, 95]. The primary acoustic mechanism by which ultrasound influenced fibrin fiber structure was thought to be cavitation, although heating and fluid streaming mechanisms may have also contributed to clot degradation [94, 106]. This work was among the first to demonstrate that ultrasound can be used to manipulate the structure of ECM proteins. Related techniques are under active investigation as therapeutic strategies to accelerate thrombolysis in animal models of ischemic stroke [107, 108].

Type I collagen is the most abundant protein in the human body, contributing to the ECMs of a variety of tissues, including skin, tendon, cornea, and bone [109] Collagen’s high abundance low antigenicity, and versatility have made it a promising starting material for the production of tissue-engineered scaffold structures in a variety of applications [110]. Under appropriate conditions in vitro, solubilized collagen can spontaneously self-assemble into 3D hydrogels, providing a valuable platform for regenerative medicine applications [44, 111]. Self-assembled collagen fibers mimic many of the features of native collagen ECM structures, including fiber diameter and periodicity [112]. However, 3D hydrogels assembled from purified collagen differ from native ECM collagen in several critical ways, including an absence of tissue-specific fiber structures and co-assembly with other ECM components [113, 114]. For this reason, numerous techniques for manipulating the structure and function of collagen within in vitro environments have been explored. Collagen polymerization parameters, including concentration, pH, and temperature, have been widely exploited to manipulate scaffold characteristics such as fiber diameter [91], pore size [115], and gel stiffness [116], and have been reviewed previously [110]. Collagen fiber characteristics directly influence cell behaviors important to wound healing, including cell spreading and adhesion, as well as migration and collagen fiber contraction [29, 115, 117, 118]. Several techniques have been developed to manipulate the hierarchical, macromolecular organization of collagen fibers within 3D hydrogels. These include the use of fluid flow [119], mechanical tension [120], electrospinning [121], and magnetic fields [122, 123] to produce aligned fibers within 3D collagen matrices. Other approaches have used PDMS or other non-adhesive molds to produce micron-scale microchannels within collagen gel structures [124].

The use of ultrasound to directly and non-invasively manipulate the structure and function of collagen hydrogels was first described by Garvin et al. [102]. This work demonstrated that ultrasound exposure (1- or 8.3-MHz, with acoustic intensities up to 30 W/cm^2^) during collagen polymerization could produce local changes in collagen fiber microstructure, including increased collagen fiber density and reduced fiber diameter [102]. Measurements of ultrasound-induced heating within the center of polymerizing gels demonstrated temperature increases of up to 10 °C above unexposed sham samples [102]. Ultrasound-induced changes in collagen fiber structure could be mimicked using a non-acoustic heat source, suggesting a thermal acoustic mechanism [102]. This conclusion is consistent with previous reports that elevated temperature during collagen polymerization is associated with thinner, more densely packed collagen fiber structures [91, 115]. One advantage of ultrasound is that local heating can be produced non-invasively and site specifically, thereby providing avenues for fabricating collagen hydrogels with spatial control of collagen fiber structure. In addition to thermal effects of ultrasound, acoustic exposure during collagen polymerization can produce collagen fiber alignment through non-thermal mechanisms [103]. This effect was only observed when ultrasound was present during the active phase of collagen fibril self-assembly [104]. The pattern of ultrasound-mediated collagen fiber alignment was consistent with simulation patterns of fluid flow within ultrasound-exposed cylindrical vessels [66, 68], and thus resembles other systems in which fluid streaming is induced by non-acoustic mechanisms, such as flow through microchannels [119].

Other groups have used laboratory benchtop sonicators to partially disrupt ECM structures. These devices operate at comparatively low frequencies (15–30 kHz), and high power (≥ 150 W total power) to induce acoustic cavitation within a fluid volume, and have been used to facilitate surface cleaning, chemical catalysis, and cell lysis [125, 126]. Examples in which benchtop sonicators have been used for regenerative medicine applications include sonication of detergent-decellularized porcine tendon, which produced fiber separation within the collagenous ECM structure [96]. Sonicated tendon scaffolds supported enhanced cellular infiltration, but did not support long-term viability of cells embedded within the center of the construct [96]. Likewise, Maller and colleagues utilized a model of sonicated type I collagen in combination with other ECM components to investigate effects on cellular morphology and integrin activation in mammary tumor cells [127]. In this study, volumes of soluble collagen were sonicated until the detection of fibers by second harmonic generation (SHG) imaging was eliminated, indicating unfolding or fragmentation of the collagen triple helical structure [127, 128]. Both examples demonstrate the feasibility of influencing collagen structure and function with ultrasound-induced bioeffects. However, typical benchtop sonicators do not offer precise control over acoustic parameters and vary among manufacturers, thereby limiting both optimization and reproducibility.

Finally, many ECM proteins contain regions that undergo conformational changes in response to temperature or mechanical force, thereby exposing new epitopes for cell and/or protein engagement [26, 89, 129]. A key example of this behavior is fibronectin, a large molecular weight glycoprotein whose incorporation into the fibrillar ECM requires application of cell-derived mechanical forces and exposure of cryptic, self-interacting epitopes [26, 130]. Interactions of fibronectin with other ECM components, including collagen, are also influenced by conformational changes that arise as a result of temperature [89] or the application of mechanical force [131]. Recent work describing the polymerization of collagen-fibronectin composite gels suggests that ultrasound exposure can trigger fibronectin fibril assembly at hydrogel surfaces [93]. Effects were observed only in the presence of ultrasound under permissive temperature conditions, suggesting that a combination of thermal and non-thermal effects of ultrasound are involved in triggering fibril formation within engineered hydrogels [93]. Collagen-fibronectin binding is one of many examples in which interactions among ECM components co-regulate downstream functions [132–134]. As investigations into effects of ultrasound expand to other composite ECM materials [101], additional examples of how ultrasound can be used to influence interactions among ECM components are likely to emerge.

### 4.2 Acoustically-Responsive Engineered Scaffolds

The development of novel techniques to enhance the susceptibility of biological systems to acoustic effects has expanded the versatility of ultrasound across both diagnostic and therapeutic domains. In particular, advances in microbubble chemistry have enabled the use of acoustically-responsive droplets as vehicles for the delivery of drugs, including recombinant proteins [135], small molecules [136], and genetic material [137]. In these systems, which vary among applications in their composition and preparation, a therapeutic payload is suspended in a cavitation-sensitive material, often gas or perfluorocarbons, and encapsulated within a stabilizing shell [138]. The development and potential applications of acoustically-responsive droplets has been reviewed recently [138, 139]. Here, we discuss specific examples of relevance to ECM-based scaffolds and within engineered environments.

A key advantage of acoustically-triggered release is its capacity to simultaneously enable spatial and temporal control over drug release [138]. Nele et al. recently reported using ultrasound to trigger calcium release from acoustically responsive liposomes to initiate fibrin hydrogel polymerization [97]. In this system, ultrasound-induced calcium release was used to activate transglutaminase activity, which in turn triggered catalysis of soluble fibrinogen to initiate hydrogel crosslinking [97]. The kinetics of calcium release and enzymatic activity, as well as the elastic modulus of the polymerized fibrin hydrogel, were sensitive to acoustic exposure duration [97]. Thus, ultrasound-induced calcium release simultaneously enabled hydrogel assembly and tuning of hydrogel mechanical properties. Many components of ECM signaling networks are sensitive to calcium, including matrix metalloprotease activation [140], integrin-mediated adhesion [141], and interactions between ECM proteins [142]. As such, techniques that take advantage of ultrasound-triggered calcium release to initiate enzymatic activity or cell signaling cascades in temporally- and spatially-defined patterns within ECM-based scaffolds are likely to expand.

Native ECM also serve as reservoirs for localized growth factor release [33]. The use of acoustically responsive droplets to mimic controlled release of growth factors has been demonstrated in fibrin gels. With this technique, growth factors are encapsulated into acoustically responsive droplets (<10 μm) which are then embedded in fibrin-based hydrogels. Exposing fibrin-embedded, basic fibroblast growth factor (bFGF)-loaded droplets to ultrasound triggered growth factor release into the surrounding media, and treatment of endothelial cells with this conditioned media enhanced cell viability [98] and vascular sprout formation [143]. Additionally, ultrasound-induced cavitation increased the porosity and stiffness of these fibrin scaffolds [98]. When similar constructs were loaded with a fluorescent tracer and implanted subcutaneously in mice, acoustic exposure supported accelerated payload release [99]. The kinetics of release were sensitive to acoustic pressure, with the composition [144] and concentration [145] of perfluorocarbons used in manufacture of acoustically responsive droplets having a significant influence on the threshold pressure at which cavitation and drug release were triggered. This technology has also been proposed as a method for sequential release of paired growth factors [146].

An alternative method to achieve ultrasound-triggered growth factor release has been to genetically engineer cells with heat-responsive gene elements. This technique exploits a well-characterized transcriptional response to heat shock [147], in which cells are transfected with plasmids containing a target gene sequence under the combined control of the HSP70B promoter and a rapamycin-dependent gene switch [148]. Thus, when engineered cells are cultured in the presence of rapamycin and exposed to high-intensity focused ultrasound (HIFU), localized heating induces expression of target genes with spatial and temporal specificity [100]. This approach has been used to induce expression of both BMP-2 [100] and VEGF [148], key growth factors for therapeutic bone fracture healing and angiogenesis, respectively [149]. Further tuning of growth factor expression has been achieved by addition of hydroxyapatite to cell-embedded fibrin gels to enhance acoustic absorption [101]. The translation potential of this approach has been demonstrated in vivo using ultrasound-induced luciferase expression in murine models [101]. Critically, the acoustic mechanisms used to induce growth factor release with heat-shock induction systems, namely absorption-dependent heating [100], are distinct from the cavitation-dependent release of growth factors from acoustically-responsive lipid droplets [98]. Thus, the secondary effects on surrounding native ECM components will likely be distinct, enabling an additional level of control in engineering custom scaffolds for specific applications.

### 4.3 Indirect Effects of Ultrasound on Cell-Mediated ECM Remodeling

A critical consideration in the fabrication of tissue engineered scaffolds is an understanding of the bi-directional relationship between the ECM and its resident cells. Within this framework, known as dynamic reciprocity, structural changes in the ECM induce changes in cell behaviors, which in turn remodel the ECM [150]. Key cellular programs associated with ECM remodeling include enhanced actin-myosin contractility and cell migration, as well as upregulation of ECM gene expression and matrix protein deposition [7]. The strategic use of intrinsic ECM-remodeling capabilities of cells has emerged as an essential tool for biomaterial fabrication. Examples include the use of geometric patterning and cell-derived tensile forces to achieve fiber alignment [27, 151, 152], and the use of tissue-specific cell types to assemble matrices with appropriate molecular composition [153, 154]. In combination, these techniques have been used to reconstruct complex biomaterials mimicking numerous features of tissue-specific ECMs, with particular utility in loadbearing tissues such as tendon [155], intervertebral discs [156] and menisci of the knee [157, 158].

Contraction of fibroblast-embedded collagen hydrogels and associated changes in cell migration and differentiation is a long-standing in vitro model for investigations of ECM remodeling in a wound-like environment [25]. Studies of fibroblast-embedded collagen hydrogels exposed to ultrasound standing wave fields during collagen polymerization showed that ultrasound exposure enhanced cell-mediated gel contraction compared to sham-exposed controls [74]. Subsequent studies demonstrated that exposing soluble collagen to ultrasound during hydrogel polymerization produces collagen fibrils that are more readily remodeled by cells than sham-exposed fibrils [102, 103]. Similar collagen remodeling was observed when primary cells derived from diabetic murine dermal explants were seeded on acoustically modified collagen hydrogels [103]. In these studies, cell-mediated collagen fibril remodeling and contraction were likely facilitated by ultrasound-induced changes in collagen structure [102, 103]. Given the role of ECM remodeling in the coordinated healing response of skin to injury [25, 159], these results raise the possibility that ultrasound-based techniques may improve current fabrication technologies for regenerative biomaterials by enhancing the healing capacity of acellular, collagen-based wound dressings.

The use of ultrasound standing wave fields as a technology to pattern cells in vitro has also emerged as a versatile technique for engineering cellular biomaterials, with applications to vascular [75–77], muscular [80, 81], and neuronal [78, 79] tissue engineering. In these systems, secondary radiation forces associated with ultrasound standing waves are used to pattern cells into planar bands or columns within solutions of soluble ECM proteins, often collagen [75] or fibrinogen [79]. Following hydrogel polymerization, cells are retained in the ultrasound-established pattern after the acoustic source is removed [160]. The majority of these studies have focused on cellular responses to acoustic patterning, including cellular morphogenesis [76] and differentiation [79]. Several pieces of evidence suggest that enhanced remodeling of the initial ECM template also contributes to cellular responses. First, vascular sprouting and collagen fiber alignment along the direction of vessel growth have been observed in acoustically-patterned systems containing vascular endothelial cells [75], consistent with observations of sprouting angiogenesis from microvessel explants [161]. Likewise, enhanced contraction of acoustically-patterned constructs has been reported in several studies [74, 80]. Critically, the direction of cell alignment can be directed to develop either parallel or perpendicular to the acoustic exposure axis by altering the orientation in which the engineered tissue is anchored [80]. Taken together, these findings suggest that the expression of tissue-mimicking cellular morphologies may arise from an enhanced capability of resident cells to remodeling the ultrasound-exposed scaffold.

## 5. Emerging Technologies

As technologies to manipulate biological systems advance, the uses of ultrasound in the fabrication of ECM-based scaffolds is also likely to expand. Many ultrasound properties are highly attractive for regenerative medicine applications, particularly the ability to be used non-invasively and with high temporal and spatial control. In this regard, ultrasound is one of several emerging modalities by which biomaterials, as well as cells and other components of the tissue-engineered environment, can be manipulated non-invasively [10]. Given the complexity of native tissues, producing engineered tissue substitutes with sufficient fidelity to restore healthy function in patients with severe disease and injury will likely require the synergy of multiple strategies and technologies. The use of tissue-specific cells to further remodel acoustically-modified ECM templates is one such example of this approach that has already generated promising preliminary success [75, 80, 103]. Here, we discuss additional examples in which ultrasound-based methodologies may extend the potential of other experimental strategies.

One technology of interest is the development of acoustic tweezing cytometry (ATC) to apply mechanical forces to cells or proteins. Pioneering work in this field demonstrated the feasibility of targeting microbubbles to the surface of cells via attachment of an integrin-binding peptide ligand to the microbubble shell [162, 163]. Exposure of microbubble-targeted cells to ultrasound induced microbubble displacement was followed by sustained generation of cellular traction forces [162, 163]. Cytoskeletal contraction required Rho- and Rho-associated kinase (ROCK)-mediated signaling [163], and was not observed when microbubbles were targeted to a non-integrin receptor [162]. Assembly of fibronectin fibrils, a key event during tissue repair [3, 23] is likewise dependent upon integrin ligation and Rho-mediated cytoskeletal contractility [130]. Thus, these studies suggest the possibility that ATC-stimulated cellular contractility may offer yet another technique to manipulate cellular ECM deposition and/or force-sensitive conformational changes to ECM proteins.

Another area of expanding potential is the use of acoustic techniques to assemble scaffold materials into increasingly complex patterns with high precision. The use of acoustic holograms, in which sound passes through a 3D-printed phase plate to generate an acoustic pressure field in a user-defined pattern, has emerged recently as a significant advance in the complexity of structures that can be achieved using ultrasound-mediated particle patterning [164]. This approach has been used to pattern cells [165] and PEG-DMA scaffolds [166] into complex geometries. The use of ultrasound to pattern PEG-based scaffolds is of particular interest to the engineering of biocompatible scaffolds, as PEG hydrogels can be functionalized with appropriate ligands to engineer specific ECM compositions and conformations [167]. Such an approach has the potential to enable the incorporation of a diverse array of bioactive signals [168], including adhesive [169, 170] and matricryptic [171] ligands, as well as enzymatically-sensitive [172] and growth factor-simulating [170, 172] sequences. Other technologies use ultrasound beam steering and vortex beams to move and steer particles in 3D through fluid materials [173]. Together, these technologies may be used in coordination to create engineered matrices of high spatial and biochemical complexity.

## 6. Conclusions

In summary, ultrasound is a versatile tool for manipulating biological systems and continues to find novel applications in the fabrication of biomaterials for regenerative medicine applications. Ultrasound has the capacity to induce several distinct effects within biological systems, including heating, fluid streaming, and microbubble cavitation. Numerous ultrasound-based techniques that have the capacity to manipulate biomaterials have emerged in recent years. These include the use of ultrasound to modify the conformation and organization of fibrillar ECM proteins either directly or indirectly, as well as innovative strategies to use ultrasound to enhance cellular activities within engineered scaffolds. The convergence of these developments with an evolving understanding of the role of ECM during tissue repair represents a significant opportunity to harness ultrasound as a non-invasive methodology for fabricating ECM-based scaffolds with enhanced complexity and regenerative capacity.

## Figures and Tables

**Table 1:** Examples of acoustic modification of ECM scaffold structure and function. Abbreviations: CW, continuous wave; USWF, ultrasound standing wave field; I_SPPA_, spatial peak, pulse average intensity; I_SPTA_, spatial peak, temporal average intensity; PFC, perfluorocarbon; rtPA, recombinant tissue plasminogen activator.

ECM/US Interaction	Acoustic Conditions	Acoustic Mechanism	Scaffold Composition	Biological Response	Reference
Direct effect on ECM proteins	8 MHz, CW I_SPPA_ ≤ 10 W/cm^2^	Thermal and non-thermal	Collagen and fibronectin	Fiber alignment, fibronectin fibril formation; Fibroblast self-assembly	[93]
Direct effect on ECM proteins	1 MHz, CW 1 – 8 W/cm^2^	Cavitation	Fibrin and rtPA	Decreased fiber density, enhanced proteolysis	[94, 95]
Direct effect on ECM proteins	Benchtop sonicator	Cavitation	Decellularized tendon	Decreased fiber density and increased pore size; Enhanced cellular infiltration	[96]
Acoustically responsive scaffold	20kHz, pulsed	Cavitation	Ca^2+^-spiked liposomes in transglutaminase-fibrinogen solution	Transglutaminase-triggered fibrinogen polymerization	[97]
Acoustically responsive scaffold	3.5 MHz, pulsed MPa +(−) = 12.9 [6.0]	Cavitation	PFC-growth factor emulsions embedded in fibrin	Increased pore size, increased stiffness; Growth factor release	[98, 99]
Acoustically responsive scaffold	2.5 MHz, CW I_SPTA_ = 658–750 W/cm^2^	Thermal	Cell-embedded fibrin (+/− hydroxyapatite)	Growth factor expression	[100, 101]
Cellular ECM Remodeling	2–2.2 MHz, USWF 0.12 MPa	USWF	Collagen	Collagen contraction; Myoblast alignment along tensional axis	[80]
Cellular ECM Remodeling	1 MHz, USWF 0.2MPa	USWF	Cell-embedded collagen	Enhanced gel contraction; Fiber alignment along vascular sprouts	[74, 75]
Direct effect on ECM proteins; Cellular ECM Remodeling	8 MHz, CW I_SPPA_ ≤ 10 W/cm^2^	Non-thermal	Collagen	Altered pore density and radial fiber alignment; Fibroblast migration and ECM contraction	[102–104]
